# Exact wave structures in magneto-optic soliton channels governed by Kudryashov-type coupled Schrödinger dynamics

**DOI:** 10.1038/s41598-026-53103-4

**Published:** 2026-05-24

**Authors:** Amany Tarek, Hamdy M. Ahmed, Niveen Badra, Islam Samir

**Affiliations:** 1 Department of Physics and Engineering Mathematics, Faculty of Engineering,Ain Shams University, Abbassia, P.O. Box 11517, Cairo, Egypt; 2 Department of Physics and Engineering Mathematics, Higher Institute of Engineering, El Shorouk Academy, El Shorouk City, Cairo, Egypt

**Keywords:** Optical solitons, Coupled nonlinear Schrödinger equation, Magneto-optic waveguides, Kudryashov-type nonlinearity, Improved Simple Equation Method (ISEM), Soliton solutions and propagation dynamicss, Mathematics and computing, Optics and photonics, Physics

## Abstract

This research establishes a comprehensive theoretical framework for soliton propagation in magneto-optic waveguides by investigating a generalized coupled Kudryashov-type nonlinear Schrödinger system under the influence of higher-order nonlinear effects. The primary objective is to resolve the complex interplay between self-steepening, nonlinear dispersion, and magnetization parameters, which are often oversimplified in standard models. By implementing the improved simple equation method, we derive a new spectrum of exact analytical solutions, including robust kink, antikink, and singular profiles, and provide their first detailed parametric characterization. Our findings demonstrate that tuning specific nonlinear coefficients allows for precise control over pulse stability and transition gradients, offering critical insights for the development of high-capacity optical communication architectures and all-optical switching technologies.

## Introduction

The study of optical solitons has emerged as a cornerstone of nonlinear optics, primarily due to their stability and potential in high-speed information transmission^1,2^. Significant research efforts have been dedicated to characterizing soliton propagation in various fiber configurations, including the analysis of optical fiber Bragg gratings and the application of Kudryashov’s generalized refractive index models^3,4^. Furthermore, the precise manipulation of soliton dynamics—such as phase-shift control in dispersion-decreasing fibers and pulse propagation in birefringent media—has been extensively documented in recent literature^5,6^. Parallel developments in mathematical physics, such as the investigation of the Kadomtsev–Petviashvili equation in fluid dynamics and the application of renormalization methods for singular perturbations, have provided a robust analytical foundation for exploring complex nonlinear wave phenomena across diverse physical systems^7,8^.

This intrinsic stability makes them highly suitable for high-speed optical communication systems. In recent years, the dynamics of solitons have been extensively explored in magneto-optic waveguides, where early studies established the potential for devices based on the interaction between magnetostatic and optical guided waves^9^. To mitigate pulse distortion and improve signal integrity, researchers have investigated the influence of spatio-temporal dispersion and cross-phase modulation^10–12^. Furthermore, a significant body of work has focused on characterizing soliton profiles under various nonlinear laws, such as dual-power, anti-cubic, and quadratic-cubic nonlinearities^13–18^. Finally, the application of generalized refractive index models, including those based on Kudryashov’s equation, has provided deep insights into soliton stability and conservation laws in magnetized media^19,20^. Consequently, the analytical study of exact soliton solutions continues to be of significant theoretical and practical interest in nonlinear optics.

Nonlinear partial differential equations (NPDEs) play a fundamental role in modeling a wide range of nonlinear phenomena in physics and engineering. Finding their exact solutions is essential for understanding the underlying dynamics, validating numerical approaches, and revealing complex nonlinear behaviors such as solitary waves and localized structures. In this regard, advanced computational frameworks, such as multi-task training strategies for phase retrieval and error compensation, are increasingly employed to ensure the robustness and accuracy of these wave analyses^21^. Many analytical methods have been developed over time for this aim, including the $\phi ^6$ model expansion^22^, the first integral method^23^, the improved simple equation method (ISEM)^24^, the modified extended direct algebraic method^25^, the general projective Riccati method^26^, the enhanced modified extended tanh function method^27^, the modified extended mapping method^28^, and Kudryashov’s method^29^. Complementing these established approaches, the Nucci reduction method^30^ and unified integration techniques^31^ have also been utilized to effectively derive robust solitary wave solutions and establish essential stability criteria for complex nonlinear models.

Furthermore, recent research has extended these frameworks to conduct bifurcation and modulational instability analyses to explore soliton formation and chaotic behaviors in nonlinear systems^32–36^. Specifically, bifurcation analysis and Riccati sub-ODE methods^37^ have provided deeper understandings of higher-order dispersion effects and chaotic wave behaviors, reinforcing the importance of such analytical frameworks. Additionally, the application of diverse integration schemes—such as the extended tanh approach, the He-Elzaki transform^38^, and the F-expansion algorithm—has provided further insights into soliton classification and dynamics within complex concatenation models^39,40^.

Recent studies have employed various mathematical techniques to obtain exact solutions for nonlinear models. For instance, the Jacobi elliptic function method has been effectively utilized to analyze soliton structures in complex media^41–43^. In parallel, researchers have applied extended rational sinh–cosh approaches to explore the dynamics of dark and singular solitons^44–46^. Furthermore, advancements in numerical and stability analysis have provided deeper insights into the pulse propagation characteristics^47^.

Among these models, the nonlinear Schrödinger equation (NLSE) plays a central role in describing a wide range of physical phenomena, including plasma waves, fluid dynamics, and optical pulse propagation in fibers^48–50^. In 2019, Kudryashov introduced a generalized nonlinear refractive index law for optical fibers, which has been successfully applied to soliton dynamics in birefringent and polarization-maintaining fibers^51^. Motivated by these developments, we consider the coupled system of the NLSE with a generalized Kudryashov-type equation in magneto-optic waveguides^52^, expressed as

1 $$\begin{aligned} & \begin{aligned}&i~u_t + i~1~u_x + b_1~u_{xx} + i~c_1~u_{xxx} + d_1~u_{xxxx} + \sigma _1~u \\&+ \Bigg (\frac{n_1}{|u|^4} + \frac{m_1}{|u|^3} + \frac{l_1}{|u|^2} + \frac{k_1}{|u|} + e_1~|u| + f_1~|u|^2 + g_1~|u|^3 + h_1~|u|^4 \Bigg ) u \\&+ \Bigg (\frac{\tau _1}{|\upsilon |^4} + \frac{\varsigma _1}{|\upsilon |^3} + \frac{\eta _1}{|\upsilon |^2} + \frac{\xi _1}{|\upsilon |} + \alpha _1~|\upsilon | + \beta _1~|\upsilon |^2 + \gamma _1~|\upsilon |^3 + \delta _1~|\upsilon |^4 \Bigg ) u \\&= Q_1~\upsilon + i \Big [\lambda _1 (|u|^2 u)_x + \nu _1 (|u|^2)_x u + \theta _1 |u|^2 u_x \Big ], \end{aligned} \end{aligned}$$

2 $$\begin{aligned} & \begin{aligned}&i~\upsilon _t + i~a_2~\upsilon _x + b_2~\upsilon _{xx} + i~c_2~\upsilon _{xxx} + d_2~\upsilon _{xxxx} + \sigma _2~\upsilon \\&+ \Bigg (\frac{n_2}{|\upsilon |^4} + \frac{m_2}{|\upsilon |^3} + \frac{l_2}{|\upsilon |^2} + \frac{k_2}{|\upsilon |} + e_2~|\upsilon | + f_2~|\upsilon |^2 + g_2~|\upsilon |^3 + h_2~|\upsilon |^4 \Bigg ) \upsilon \\&+ \Bigg (\frac{\tau _2}{|u|^4} + \frac{\varsigma _2}{|u|^3} + \frac{\eta _2}{|u|^2} + \frac{\xi _2}{|u|} + \alpha _2~|u| + \beta _2~|u|^2 + \gamma _2~|u|^3 + \delta _2~|u|^4 \Bigg ) \upsilon \\&= Q_2~u + i \Big [\lambda _2 (|\upsilon |^2 \upsilon )_x + \nu _2 (|\upsilon |^2)_x \upsilon + \theta _2 |\upsilon |^2 \upsilon _x \Big ]. \end{aligned} \end{aligned}$$

Here, *u*(*x*, *t*) and $\upsilon (x,t)$ denote the wave profiles, and all constants are real for $j = 1,2$. The coefficients $a_j, b_j, c_j,$ and $d_j$ correspond to dispersion terms, including intermodal dispersion, chromatic dispersion, third-order dispersion, and fourth-order dispersion, respectively. The parameters $n_j, m_j, l_j, k_j, e_j, f_j, g_j, h_j$ arise from self-phase modulation, while $\tau _j, \varsigma _j, \eta _j, \xi _j, \alpha _j, \beta _j, \gamma _j, \delta _j$ describe cross-phase modulation. The terms $\sigma _j$ represent detuning, $\lambda _j, \nu _j, \theta _j$ account for self-steepening and nonlinear dispersion effects, and $Q_j$ are the magneto-optic coupling parameters.

This coupled NLSE–Kudryashov system incorporates higher-order nonlinear effects, including self-steepening, Kerr-type responses, and magnetization^53,54^, providing a more comprehensive model for soliton dynamics in magneto-optic fibers. It is closely related to other extensions of the NLSE, such as derivative forms capturing Raman scattering^55^, fractional versions modeling long-range dispersion^56^, and cubic–quintic equations describing competing nonlinearities^57,58^. Furthermore, phenomena such as chirped solitons in metamaterials^59^, the Fokas–Lenells equation^60^, derivative NLSE^61^, and soliton propagation in negative-index media^62^ naturally emerge within this framework, which admits a rich variety of solutions including singular, antikink-type, kink-type solitons, rational, singular periodic, and exponential solutions^63,64^. Beyond optical applications, this model is also relevant to plasma waves^65^, Bose–Einstein condensates^66^, and fluid dynamics^67^, highlighting its broad significance in the study of nonlinear wave phenomena^68^, as well as in oceanic wave propagation and maritime transport systems^69^. Furthermore, recent studies have extended this framework to analyze the impact of stochastic effects and noise influences on coupled wave systems, providing critical insights into energy flow and turbulence dynamics in plasma environments^70,71^

The selection of the Kudryashov-type coupled Schrödinger system in this study is motivated by several critical factors that transcend the limitations of the standard NLSE. Firstly, this model is particularly significant in the context of magneto-optic waveguides, where the interplay between electromagnetic fields and magnetized media requires a more robust mathematical framework. Unlike the standard NLSE, the Kudryashov-type model incorporates higher-order nonlinear effects, such as self-steepening and nonlinear dispersion, which are essential for describing the propagation of ultra-short pulses in high-speed optical fiber systems. Furthermore, the coupled nature of the equations allows for the investigation of complex interactions between two co-propagating waves, providing a more realistic representation of signal transmission.

Despite the extensive literature on optical solitons, existing studies have primarily focused on the standard NLSE or its simpler variations. While valuable, these models often fail to account for the complex interaction between Kudryashov-type nonlinearities, higher-order dispersion, and self-steepening effects in magneto-optic waveguides. Consequently, there remains a critical need for a more comprehensive model and an efficient analytical framework that can accurately predict and analyze these diverse wave phenomena in high-performance optical communication architectures.

In light of these research gaps, the primary contributions of this study are threefold: (i) it presents a systematic analytical investigation of the coupled NLSE–Kudryashov system in the context of magneto-optic waveguides; (ii) it employs the improved simple equation method (ISEM) to derive a comprehensive spectrum of soliton solutions—including kink, antikink, and singular profiles—which are often overlooked in standard NLSE analyses; and (iii) it provides a detailed physical discussion of how higher-order nonlinear effects and magnetization parameters influence pulse stability.

To acquire these precise solutions, the ISEM is employed throughout this study. The remainder of the paper is organized as follows: Section "The present approach" provides a detailed description of the ISEM. In Section "Results of the study", the method is applied to analyze the coupled system given by Equations (1) and (2). Section "Graphical representation of the soliton solutions" presents graphical illustrations of selected solutions in both two and three dimensions. Section "Results and discussion" discusses the physical significance of the derived solutions and explores how variations in the system’s parameters influence the dynamic behavior and profiles of the obtained solitons. Ultimately, the research is concluded with a summary of the main findings and a discussion of potential directions for future work.

## The present approach

This section provides a summary of the ISEM^7^.

The ISEM offers distinct advantages in the context of solving complex NPDEs. Compared to traditional analytical techniques, such as the $\phi ^6$ model expansion, the first integral method, or standard Kudryashov’s method, the ISEM provides a more streamlined computational framework. While many existing methods often require lengthy algebraic calculations or specific constraint conditions that may restrict the variety of obtained solutions, the ISEM is highly efficient in extracting a broader spectrum of localized wave structures—including singular, kink, and antikink solitons—without the need for complex transformations. Furthermore, its ability to handle high-order nonlinearities, such as those present in the Kudryashov-type coupled system, makes it a robust tool for exploring intricate wave dynamics in magneto-optic waveguides.

Assuming the following NLPDE:

3 $$\begin{aligned} K(U, U_t, U_x, U_{xx}, U_{tx}, \dots )=0, \end{aligned}$$

where *K* is a polynomial in the unknown function *U*(*x*, *t*) and its partial derivatives. To find solutions of Eqn. (3) via the ISEM, the following main procedures must be implemented:

**Step (1)**: By executing this transformation, an ordinary differential equation (ODE) is created using Eqn. (3):

4 $$\begin{aligned} U(x,t)=U(\mu ), \quad \mu =x-Ct, \quad C\ne 0, \end{aligned}$$

where *C* is the traveling wave velocity. In Eqn. (3), the NLPDE then transforms into:

5 $$\begin{aligned} Y(U, U', U'', U''', \dots )=0. \end{aligned}$$

**Step (2)**: Using this proposed method, the solution to Eqn. (5) takes the following form:

6 $$\begin{aligned} U(\mu )=\Psi _0+\sum _{j=1}^{N}{\left( \Psi _j Z^j(\mu )+\Phi _{j} Z^{-j}(\mu )\right) }, \end{aligned}$$

where $Z(\mu )$ fulfills the following differential equation:

7 $$\begin{aligned} Z'(\mu )=D_0+D_1 Z(\mu )+D_2 Z^2(\mu ). \end{aligned}$$

**Step (3)**: Determine the balance number *N* in Eqn. (6) from Eqn. (5). To find the integer *N* in the proposed solution, use the homogeneous balance principle. In the simplified ODE, identify the highest-order derivative term and the highest-order nonlinear term, then balance the degrees of these terms to determine the value of *N*.

**Step (4)**: The system of nonlinear equations is created by setting the coefficients of $Z^j(\mu )$ to equal zero for $j=0, 1, 2, \dots$ and then substituting the suggested solution from Eqn. (6) and Eqn. (7) into Eqn. (5).

**Step (5)**: Mathematica or Maple software tools can be used to solve the nonlinear system of equations created in Step (4) and to obtain the unknown values $\Psi _0, \Psi _1, \Phi _1$ and *C*.

**Step (6)**: Eqn. (7) can produce a range of solutions depending on the values of $D_0, D_1,$ and $D_2$:

**First Family:** $$D_2=0$$

$$\begin{aligned} Z(\mu )=\frac{e^{D_1 ~\mu }}{D_1}-\frac{D_0}{D_1}. \end{aligned}$$

**Second Family:** $$D_1=0$$

$$\begin{aligned} Z(\mu )=-\sqrt{-\frac{D_0}{D_2}} \tanh (\sqrt{-D_0~ D_2}~ \mu ), \quad D_0 D_2 < 0 \end{aligned}$$

$$\begin{aligned} Z(\mu )=\sqrt{\frac{D_0}{D_2}} \tan (\sqrt{D_0~ D_2}~ \mu ), \quad D_0 D_2> 0 \end{aligned}$$

**Third Family:** $$D_0=0$$

$$\begin{aligned} Z(\mu )=\frac{D_1 e^{D_1~ \mu }}{1-D_2~ e^{D_1~ \mu }}, \quad Z(\mu )=\frac{-D_1 e^{D_1 ~\mu }}{1+D_2 ~e^{D_1 ~\mu }}. \end{aligned}$$

**Step (7)**: The final stage involves substituting the general solution of Eqn. (7) and the resultant unknowns into the proposed method of Eqn. (6) in order to obtain distinct solutions for the simulation under investigation.

**Fig. 1:**
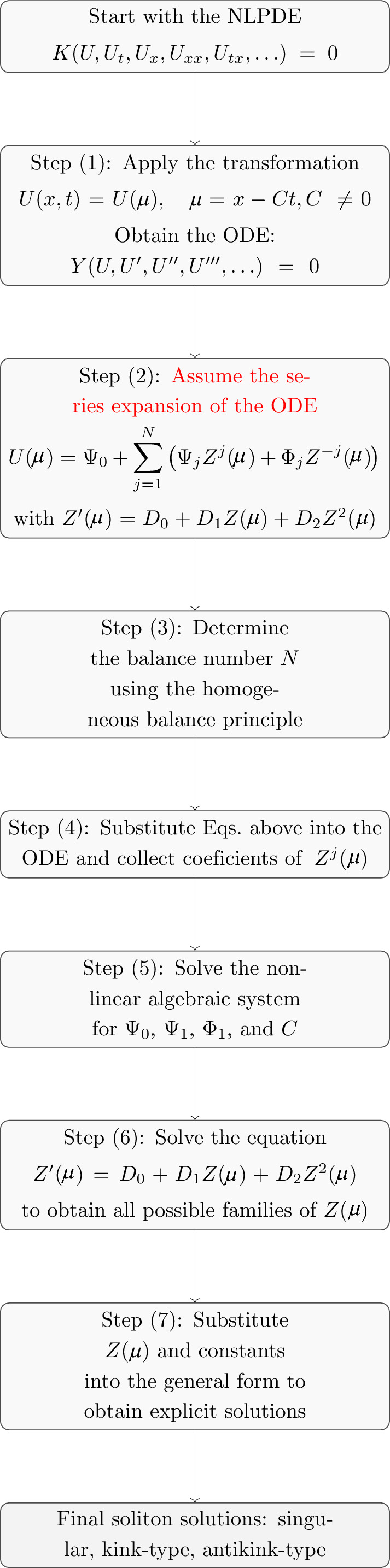
Flowchart summarizing the ISEM.

To provide a clearer overview of the analytical procedure adopted in this study, we summarize the main steps used to derive the obtained soliton solutions. Figure 1 presents a flowchart that outlines the systematic sequence leading from the transformed resonant NLSE to the explicit forms of the antikink-type, kink-type, and singular solitons.

The ISEM for finding soliton solutions offers significant advantages over other techniques. Unlike methods that require solving an auxiliary equation or rely on predefined functions, the ISEM is generally more straightforward and computationally efficient. Table 1 provides a comprehensive comparison between the ISEM and other analytical methods, highlighting its distinctive features and performance. It is known for its ability to produce a wider variety of exact traveling wave solutions, often uncovering solutions that other methods miss. Furthermore, the solutions derived from the ISEM typically include free parameters, allowing for a flexible and comprehensive exploration of a system’s behavior by generating different types of solitary wave solutions, such as kink-type, antikink-type, and singular forms, from a single general solution. This combination of simplicity, efficiency, and flexibility makes the ISEM a powerful and reliable tool for researchers in mathematical physics. However, the ISEM also has certain limitations compared to other analytical techniques. In some cases, it may involve tedious algebraic manipulation when dealing with highly complex nonlinearities, and it often requires careful selection of parameter constraints to ensure the physical relevance and stability of the obtained solutions.

**Table 1:** Comparison of the Improved Simple Equation Method (ISEM) with other common analytical techniques.

Method	Complexity	Solution Variety	Complex Transf.	High-Order Nonlinearities
ISEM (Proposed)	Streamlined	Broad (Kink, Antikink, Singular)	Minimal	Highly Robust
Improved Mod. Tanh Function	Moderate	High	Minimal	Moderate
Mapping Method	Moderate	Good	Minimal	Moderate
$\phi ^6$-Model Expansion	High	Restricted	Required	Moderate
First Integral Method	Moderate	Specific	Required	Limited
Standard Kudryashov	Low	Moderate	Minimal	Limited
Jacobi Elliptic Method	Moderate	Periodic/Solitonic	Complex	Moderate

## Results of the study

This section aims to provide the following form of solutions for the system of equations (1) and (2).

8 $$\begin{aligned} \begin{aligned}&u(x,t)=H_1(\mu )~ e^{i(t~\omega +\Theta -\kappa ~ x)},\\&\upsilon (x,t)=H_2(\mu )~ e^{i(t~\omega +\Theta -\kappa ~ x)},~ \mu =x-C ~t, \end{aligned} \end{aligned}$$

,where $\Theta$, *C*, $\omega$, $\kappa$ represent phase constant, soliton speed, wave number and soliton frequency respectively. Using Eqn. (8) After applying Eqn. (7) to Eqn. (1) and splitting the result into real and imaginary halves, we obtain:

The real component:

9 $$\begin{aligned} \begin{aligned} \Re _1 :\;&k_1+\frac{n_1}{H_1{}^3}+\frac{m_1}{H_1{}^2}+\frac{l_1}{H_1}-\omega ~ H_1+\kappa ~a_1~ H_1-\kappa ^2~ b_1~ H_1-\kappa ^3~ c_1~ H_1+\kappa ^4 ~d_1 ~H_1\\&+\sigma _1 ~H_1+e_1~ H_1{}^2+f_1~ H_1{}^3-\kappa ~\theta _1~ H_1{}^3-\kappa ~\lambda _1~ H_1{}^3+g_1~ H_1{}^4+h_1~H_1{}^5\\&+\frac{\tau _1 ~H_1}{H_2{}^4}+\frac{\varsigma _1 ~H_1}{H_2{}^3}+\frac{\eta _1 ~H_1}{H_2{}^2}+\frac{\xi _1 ~H_1}{H_2}-Q_1 ~H_2+\alpha _1~ H_1~ H_2+\beta _1~ H_1~ H_2{}^2\\&+\gamma _1~ H_1 ~H_2{}^3+\delta _1~ H_1 ~H_2{}^4+b_1 ~H_1''+3~ \kappa ~ c_1~ H_1''-6~ \kappa ^2~ d_1 ~H_1''+d_1~ H_1{}^{(4)}=0,\\ \Re _2:\;&k_2-Q_2~ H_1+\frac{n_2}{H_2{}^3}+\frac{m_2}{H_2{}^2}+\frac{l_2}{H_2}-\omega ~ H_2+\kappa ~ a_2 ~H_2-\kappa ^2~ b_2~ H_2-\kappa ^3~ c_2 ~H_2+\kappa ^4~ d_2~ H_2\\&+\sigma _2~ H_2+\frac{\tau _2 ~H_2}{H_1{}^4}+\frac{\varsigma _2~ H_2}{H_1{}^3}+\frac{\eta _2 ~H_2}{H_1{}^2}+\frac{\xi _2~ H_2}{H_1}+\alpha _2~ H_1~ H_2+\beta _2 ~H_1{}^2~ H_2+\gamma _2 ~H_1{}^3~ H_2\\&+\delta _2 ~H_1{}^4~ H_2+e_2~ H_2{}^2+f_2~ H_2{}^3-\kappa ~\theta _2 ~H_2{}^3-\kappa ~\lambda _2 ~H_2{}^3+g_2~ H_2{}^4+h_2~ H_2{}^5+b_2~ H_2''\\&+3 ~\kappa ~c_2~ H_2''-6 ~\kappa ^2~ d_2~ H_2''+d_2~ H_2{}^{(4)}=0. \end{aligned} \end{aligned}$$

The imaginary components:

10 $$\begin{aligned} \begin{aligned} \Im _1:\;&-C H_1'+a_1~ H_1'-2 ~\kappa ~b_1 ~H_1'-3 ~\kappa ^2~ c_1 ~H_1'+4 ~\kappa ^3 ~d_1 ~H_1'-\theta _1 ~H_1{}^2~ H_1'-3 ~\lambda _1~ H_1{}^2 ~H_1'\\&-2~ \nu _1~ H_1{}^2~ H_1'+c_1~ H_1{}^{(3)}-4 ~\kappa ~d_1~H_1{}^{(3)}=0,\\ \Im _2:\;&-C~ H_2'+a_2~ H_2'-2~ \kappa ~ b_2 ~H_2'-3~ \kappa ^2~ c_2 ~H_2'+4 ~\kappa ^3 ~d_2~ H_2'-\theta _2~ H_2{}^2 ~H_2'-3~\lambda _2 ~H_2{}^2~ H_2'\\&-2~ \nu _2~ H_2{}^2~ H_2'+c_2~ H_2{}^{(3)}-4~ \kappa ~ d_2 ~H_2{}^{(3)}=0. \end{aligned} \end{aligned}$$

Setting Eqn. (10)’s coefficient to zero yields

11 $$\begin{aligned} \begin{aligned}&\kappa =\frac{c_1}{4d_1}=\frac{c_2}{4d_2},\\&C=a_1-2 ~\kappa ~ b_1-3~ k^2~ c_1+4~ k^3~ d_1=a_2-2~ \kappa ~ b_2-3 ~k^2~ c_2+4~ k^3~ d_2,\\&\theta _1 +3~ \lambda _1 +2 ~\nu _1=0, \\&\theta _2 +3~ \lambda _2 +2~ \nu _2=0. \end{aligned} \end{aligned}$$

Set

12 $$\begin{aligned} H_2(\mu )=\rho ~H_1(\mu ),~\rho \ne 0~or~1. \end{aligned}$$

So Eqn. (9) transform into

13 $$\begin{aligned} \begin{aligned} \Re _1:\;&k_1+\frac{n_1}{H_1{}^3}+\frac{m_1}{H_1{}^2}+\frac{l_1}{H_1}-\rho ~ Q_1 ~H_1+\left( -\omega +\kappa ~ a_1-\kappa ^2~ b_1-\kappa ^3~ c_1+\kappa ^4~ d_1+\sigma _1\right) ~ H_1+e_1 ~H_1{}^2\\&+\left( f_1-\kappa ~ \left( \theta _1+\lambda _1\right) \right) ~H_1{}^3+g_1~ H_1{}^4+h_1~ H_1{}^5\\&+H_1~ \left( \frac{\tau _1}{\rho ^4~ H_1{}^4}+\frac{\varsigma _1}{\rho ^3 ~H_1{}^3}+\frac{\eta _1}{\rho ^2~ H_1{}^2}+\frac{\xi _1}{\rho ~ H_1}+\rho ~ \alpha _1 ~H_1+\rho ^2 ~\beta _1~ H_1{}^2+\rho ^3 ~\gamma _1~ H_1{}^3+\rho ^4 ~\delta _1 ~H_1{}^4\right) \\&+\left( b_1+3 ~\kappa ~c_1-6 ~\kappa ^2 ~d_1\right) ~ H_1''+d_1~ H_1{}^{(4)}=0,\\ \Re _2:\;&k_2+\frac{n_2}{\rho ^3~ H_1{}^3}+\frac{m_2}{\rho ^2~ H_1{}^2}+\frac{l_2}{\rho ~H_1}-Q_2~ H_1+\rho ~ \left( -\omega +\kappa ~ a_2-\kappa ^2~ b_2-\kappa ^3 ~c_2+\kappa ^4~ d_2+\sigma _2\right) ~H_1\\ &+\rho ^2~ e_2~ H_1{}^2+\rho ^3 ~\left( f_2-\kappa ~\left( \theta _2+\lambda _2\right) \right) H_1{}^3+\rho ^4~ g_2 ~H_1{}^4+\rho ^5~ h_2~ H_1{}^5\\&+\rho ~H_1 \left( \frac{\tau _2}{H_1{}^4}+\frac{\varsigma _2}{H_1{}^3}+\frac{\eta _2}{H_1{}^2}+\frac{\xi _2}{H_1}+\alpha _2 ~H_1+\beta _2~ H_1{}^2+\gamma _2~ H_1{}^3+\delta _2~ H_1{}^4\right) \\&+\rho ~\left( b_2+3 ~\kappa ~ c_2-6 ~\kappa ^2 ~d_2\right) ~ H_1''+\rho ~ d_2~ H_1{}^{(4)}=0 \end{aligned} \end{aligned}$$

By comparing the coefficients of the same term in the system of equations in (13), we find

14 $$\begin{aligned} \begin{aligned}&k_1=k_2,~n_1=\frac{n_2}{\rho ^3},~m_1=\frac{m_2}{\rho ^2},~l_1=\frac{l_2}{\rho },~Q_1~\rho =Q_2,\\&\left( -\omega +\kappa ~ a_1-\kappa ^2~ b_1-\kappa ^3 ~c_1+\kappa ^4~ d_1+\sigma _1\right) =\rho ~ \left( -\omega +\kappa ~ a_2-\kappa ^2~ b_2-\kappa ^3 ~c_2+\kappa ^4~ d_2+\sigma _2\right) , \\&e_1=\rho ^2~ e_2,~\left( f_1-\kappa ~\left( \theta _1+\lambda _1\right) \right) =\rho ^3 ~\left( f_2-\kappa ~\left( \theta _2+\lambda _2\right) \right) ,~g_1=\rho ^4~ g_2,~ h_1=\rho ^5~ h_2,\\&\left( \frac{\tau _1}{\rho ^4}+\frac{\varsigma _1}{\rho ^3}+\frac{\eta _1}{\rho ^2}+\frac{\xi _1}{\rho }+\rho ~ \alpha _1+\rho ^2 ~\beta _1+\rho ^3 ~\gamma _1+\rho ^4 ~\delta _1 \right) =\rho ~ \left( \tau _2+\varsigma _2+\eta _2+\xi _2+\alpha _2 +\beta _2+\gamma _2+\delta _2\right) ,\\&\left( b_1+3 ~\kappa ~ c_1-6 ~\kappa ^2 ~d_1\right) =\rho ~\left( b_2+3 ~\kappa ~ c_2-6 ~\kappa ^2 ~d_2\right) ,~d_1=\rho ~ d_2. \end{aligned} \end{aligned}$$

By multiplying Eqn. (13) part $\Re _1$ by $H_1(\mu )^3$ and replacing the constants in Eqn. (14), the following main NPDE generate:

15 $$\begin{aligned} \begin{aligned}&H_1{}^3~H_1{}^{(4)}+A_0~H_1{}^3 ~ H_1''+A_1~ H_1+A_2~H_1{}^2+A_3~ H_1{}^3+A_4~ H_1{}^4+A_5~ H_1{}^5+A_6~ H_1{}^6\\&+A_7~ H_1{}^7+A_8~H_1{}^8+R=0 \end{aligned} \end{aligned}$$

Where,

$$\begin{aligned} A_0=\left( b_1+3 ~\kappa ~ c_1-6 ~\kappa ^2 ~d_1\right) ,~A_1=m_1+\frac{\varsigma _1}{\rho ^3},~A_2=\frac{\eta _1}{\rho ^2}+l_1, \end{aligned}$$

$$A_3=k_1+\frac{\xi _1}{\rho },~A_4=-\omega +\kappa ~ a_1-\kappa ^2~ b_1-\kappa ^3 ~c_1+\kappa ^4~ d_1+\sigma _1-\rho ~Q_1,$$

$$\begin{aligned} A_5=e_1+\rho ~\alpha _1,~A_6=f_1-\kappa ~\left( \theta _1+\lambda _1\right) +\rho ^2~\beta _1, \end{aligned}$$

$$\begin{aligned} A_7=g_1+\rho ^3~\gamma _1,~A_8=h_1+\rho ^4~\delta _1,~R=n_1+\frac{\tau _1}{\rho ^4}. \end{aligned}$$

The integer *N* must be obtained by applying the balance principle in order to apply the ISEM to Eqn. (15). The result is $N=1$ when the $H_1{}^3~H_1{}^{(4)}$ term and the $H_1{}^8$ term are balanced. Using the suggested method, the answer to Eqn. (15) will be stated in the following format.

16 $$\begin{aligned} H_1(\mu )=\Psi _0+\Psi _1~ Z(\mu )+\Phi _1 ~Z(\mu )^{-1}. \end{aligned}$$

Substituting by Eqn. (16) and its Riccati equation Eqn. (7) in Eqn. (15). After then, all of the factors of $Z^i$ will be equal to zero, creating a system of non-linear equations that can be solved with Mathematica software. The outcomes are as follows:

**Case 1**. $$D_2=0$$

**Result(1)**

17 $$\begin{aligned} \begin{aligned}&\Psi _1=0,~A_1=0,~A_2=0,\\&A_3=\frac{\Psi _0~ \left( 2 ~D_0^2 ~\Psi _0^2-3~ D_0~ D_1 ~\Psi _0 ~\Phi _1+D_1^2~ \Phi _1^2\right) \left( 12 ~D_0^2 ~\Psi _0^2-12~ D_0~ D_1 ~\Psi _0 ~~\Phi _1+A_0 \Phi _1^2+D_1^2~ \Phi _1^2\right) }{\Phi _1^4},\\&A_4=\frac{1}{\Phi _1^4}\left( -120 ~D_0^4~ \Psi _0^4+240 ~D_0^3~ D_1 ~\Psi _0^3 ~\Phi _1-6~ A_0~ D_0^2 ~\Psi _0^2 ~\Phi _1^2-150 ~D_0^2~ D_1^2~ \Psi _0^2 ~\Phi _1^2\right. \\&\left. +6 ~A_0 ~D_0 ~D_1 ~\Psi _0 ~\Phi _1^3+30 ~D_0 ~D_1^3~ \Psi _0~ \Phi _1^3-A_0~ D_1^2~ \Phi _1^4-D_1^4~ \Phi _1^4\right) ,\\&A_5=-\frac{3~ D_0~ \left( -2~ D_0 ~\Psi _0+D_1 ~\Phi _1\right) ~\left( 40 ~D_0^2~ \Psi _0^2-40~ D_0 ~D_1 ~\Psi _0 ~\Phi _1+A_0~ \Phi _1^2+5~ D_1^2~ \Phi _1^2\right) }{\Phi _1^4},\\&A_6=-\frac{2~ D_0^2 ~\left( 120~ D_0^2 ~\Psi _0^2-120~ D_0~ D_1 ~\Psi _0~ \Phi _1+A_0 ~\Phi _1^2+25 ~D_1^2 ~\Phi _1^2\right) }{\Phi _1^4},\\&\Psi _0=\frac{\Phi _1~ \left( 60 ~D_0^3 ~D_1+A_7~ \Phi _1^3\right) }{120~ D_0^4}, ~ \Phi _1=-\frac{(-3)^{1/4} ~2^{3/4}~ D_0}{A_8^{1/4}},~R=0. \end{aligned} \end{aligned}$$

Thus, the corresponding singular soliton solutions is obtained in the following form:

18 $$\begin{aligned} \begin{aligned}&u(x,t)=\left( -\frac{(-3)^{1/4} ~2^{3/4} ~D_0}{A_8^{1/4}~ \left( \frac{e^{(-C t+x)~ D_1}}{D_1}-\frac{D_0}{D_1}\right) }-\frac{\left( -\frac{1}{2}\right) ^{1/4} \left( -\frac{4 ~(-3)^{3/4}~ 2^{1/4}~ A_7 ~D_0^3}{A_8^{3/4}}+60~ D_0^3~ D_1\right) }{20~ 3^{3/4}~ A_8^{1/4}~ D_0^3}\right) ~e^{i(t ~\omega +\Theta -\kappa ~x)},\\&\upsilon (x,t)=\left( - \frac{(-3)^{1/4} ~2^{3/4} ~D_0}{A_8^{1/4}~ \left( \frac{e^{(-C t+x)~ D_1}}{D_1}-\frac{D_0}{D_1}\right) }-\frac{\left( -\frac{1}{2}\right) ^{1/4} \left( -\frac{4 ~(-3)^{3/4}~ 2^{1/4}~ A_7 ~D_0^3}{A_8^{3/4}}+60~ D_0^3~ D_1\right) }{20~ 3^{3/4}~ A_8^{1/4}~ D_0^3}\right) ~\rho ~e^{i(t ~\omega +\Theta -\kappa ~x)},\\&~~D_0>0,~D_1>0,~A_7>0,~A_8<0. \end{aligned} \end{aligned}$$

**Case 2**. $$D_1=0$$

**Result(1)**

19 $$\begin{aligned} \begin{aligned}&\Psi _1=0,~A_1=0,~A_2=0,\\&A_3=\frac{2~D_0~\Psi _0~\left( D_0~\Psi _0^2+D_2~\Phi _1^2\right) ~\left( 12~D_0^2~\Psi _0^2+A_0~\Phi _1^2+8~D_0~D_2~\Phi _1^2\right) }{\Phi _1^4},\\&A_4=\frac{2~\left( 60~D_0^4~\Psi _0^4+3~A_0~D_0^2~\Psi _0^2~\Phi _1^2+60~D_0^3~D_2~\Psi _0^2~\Phi _1^2+A_0~D_0~D_2~\Phi _1^4+8~D_0^2 D_2^2~\Phi _1^4\right) }{\Phi _1^4},\\&A_5=\frac{6~D_0^2~\Psi _0~\left( 40~D_0^2~\Psi _0^2+A_0~\Phi _1^2+20~D_0~D_2~\Phi _1^2\right) }{\Phi _1^4},\\&A_6=-\frac{2~\left( 120~D_0^4~\Psi _0^2+A_0~D_0^2~\Phi _1^2+20~D_0^3~D_2~\Phi _1^2\right) }{\Phi _1^4},\\&\Psi _0=-\frac{A_7}{5~A_8},~\Phi _1=\frac{(-3)^{1/4}~2^{3/4}~D_0}{A_8^{1/4}},~R=0. \end{aligned} \end{aligned}$$

Thus, the corresponding singular soliton solutions are obtained in the following form:

20 $$\begin{aligned} \begin{aligned}&u(x,t)=\left( -\frac{A_7}{5~A_8}-\frac{(-3)^{1/4}~2^{3/4}~\text {Coth}\left[ \sqrt{-(-C~t+x)~D_0~D_2}\right] ~D_0}{A_8^{1/4}~\sqrt{-\frac{D_0}{D_2}}}\right) ~e^{i(t ~\omega +\Theta -\kappa ~x)},\\&\upsilon (x,t)=\left( - \frac{A_7}{5~A_8}-\frac{(-3)^{1/4}~2^{3/4}~\text {Coth}\left[ \sqrt{-(-C~t+x)~D_0~D_2}\right] ~D_0}{A_8^{1/4}~\sqrt{-\frac{D_0}{D_2}}}\right) ~\rho ~e^{i(t ~\omega +\Theta -\kappa ~x)},\\&~D_0~D_2<0,~A_8<0. \end{aligned} \end{aligned}$$

Thus, the corresponding singular periodic solutions are obtained in the following form:

21 $$\begin{aligned} \begin{aligned}&u(x,t)=\left( -\frac{A_7}{5~A_8}-\frac{(-3)^{1/4}~2^{3/4}~\text {Cot}\left[ \sqrt{(-C~t+x)~D_0~D_2}\right] ~D_0}{A_8^{1/4}~\sqrt{\frac{D_0}{D_2}}}\right) ~e^{i(t ~\omega +\Theta -\kappa ~x)},\\&\upsilon (x,t)=\left( -~ \frac{A_7}{5~A_8}-\frac{(-3)^{1/4}~2^{3/4}~\text {Cot}\left[ \sqrt{(-C~t+x)~D_0~D_2}\right] ~D_0}{A_8^{1/4}~\sqrt{\frac{D_0}{D_2}}}\right) ~\rho ~e^{i(t ~\omega +\Theta -\kappa ~x)},\\&~~D_0~D_2>0,~A_8<0. \end{aligned} \end{aligned}$$

**Result(2)**

22 $$\begin{aligned} \begin{aligned}&~\Phi _1=0,~D_1=0,~A_1=0,~A_2=0\\&A_3=\frac{2~D_2~\Psi _0~\left( D_2~\Psi _0^2+D_0~\Psi _1^2\right) ~\left( 12~D_2^2~\Psi _0^2+A_0~\Psi _1^2+8~D_0~D_2~\Psi _1^2\right) }{\Psi _1^4},\\&A_4=-\frac{2~\left( 60~D_2^4~\Psi _0^4+3~A_0~D_2^2~\Psi _0^2~\Psi _1^2+60~D_0~D_2^3~\Psi _0^2~\Psi _1^2+A_0~D_0~D_2~\Psi _1^4+8~D_0^2 D_2^2~\Psi _1^4\right) }{\Psi _1^4},\\&A_5=\frac{6~D_2^2~\Psi _0~\left( 40~D_2^2~\Psi _0^2+A_0~\Psi _1^2+20~D_0~D_2~\Psi _1^2\right) }{\Psi _1^4},\\&A_6=-\frac{2~\left( 120~D_2^4~\Psi _0^2+A_0~D_2^2~\Psi _1^2+20~D_0~D_2^3~\Psi _1^2\right) }{\Psi _1^4},\\&\Psi _0=-\frac{A_7}{5~A_8},~\Psi _1=\frac{(-3)^{1/4}~2^{3/4}~D_2}{A_8^{1/4}},~R=0. \end{aligned} \end{aligned}$$

Thus, the corresponding antikink-type soliton solutions are obtained in the following form:

23 $$\begin{aligned} \begin{aligned}&u(x,t)=\left( -\frac{A_7}{5~A_8}-\frac{(-3)^{1/4}~2^{3/4}~\sqrt{-\frac{D_0}{D_2}}~D_2~\text {Tanh}\left[ (-C~t+x)~\sqrt{-D_0~D_2}\right] }{A_8^{1/4}}\right) ~e^{i(t ~\omega +\Theta -\kappa ~x)},\\&\upsilon (x,t)=\left( -~\frac{A_7}{5~A_8}-\frac{(-3)^{1/4}~2^{3/4}~\sqrt{-\frac{D_0}{D_2}}~D_2~\text {Tanh}\left[ (-C~t+x)~\sqrt{-D_0~D_2}\right] }{A_8^{1/4}}\right) ~\rho ~e^{i(t ~\omega +\Theta -\kappa ~x)},\\&~~D_0~D_2<0,~A_8<0. \end{aligned} \end{aligned}$$

Thus, the corresponding periodic solutions are obtained in the following form:

24 $$\begin{aligned} \begin{aligned}&u(x,t)=\left( -\frac{A_7}{5~A_8}-\frac{(-3)^{1/4}~2^{3/4}~\sqrt{\frac{D_0}{D_2}}~D_2~\text {Tan}\left[ (-C~t+x)~\sqrt{D_0~D_2}\right] }{A_8^{1/4}}\right) ~e^{i(t ~\omega +\Theta -\kappa ~x)},\\&\upsilon (x,t)=\left( -~\frac{A_7}{5~A_8}-\frac{(-3)^{1/4}~2^{3/4}~\sqrt{\frac{D_0}{D_2}}~D_2~\text {Tan}\left[ (-C~t+x)~\sqrt{D_0~D_2}\right] }{A_8^{1/4}}\right) ~\rho ~e^{i(t ~\omega +\Theta -\kappa ~x)},\\&~~D_0~D_2>0,~A_8<0. \end{aligned} \end{aligned}$$

**Case 3**. $$D_0=0$$

**Result(1)**

25 $$\begin{aligned} \begin{aligned}&\Phi _1=0,~D_0=0,~A_1=0,~A_2=0,~A_3=0,~A_4=\frac{2~\left( -8~D_2^4~\alpha _0^4+A_0~D_2^2~\Psi _0^2~\Psi _1^2\right) }{\Psi _1^4},\\&~A_5=0,~A_6=-\frac{2~D_2^2~\left( 120~D_2^2~\Psi _0^2-120~D_1~D_2~\Psi _0~\Psi _1+A_0~\Psi _1^2+25~D_1^2~\Psi _1^2\right) }{\Psi _1^4},\\&A_8=-\frac{24~D_2^4}{\Psi _1^4},~\Psi _0=\frac{\sqrt{\frac{3}{5}}~\sqrt{-A_6+\sqrt{\frac{-~A_0~\sqrt{A_8}}{\sqrt{6}}}}}{\sqrt{A_8}},~\Psi ~_1=\frac{2~D_2~\alpha _0}{D_1},~R=0. \end{aligned} \end{aligned}$$

Thus, the corresponding Kink-type soliton solutions are obtained in the following form:

26 $$\begin{aligned} \begin{aligned}&u(x,t)=\left( -\frac{\sqrt{-6~A_6+6^{3/4}~\sqrt{-A_0~\sqrt{A_8}}}~\left( 1+e^{(-C~t+x)~D_1}~D_2\right) }{\sqrt{10}~\sqrt{A_8}~\left( -1+e^{(-C~t+x) D_1}~D_2\right) }\right) ~e^{i(t ~\omega +\Theta -\kappa ~x)},\\&\upsilon (x,t)=\left( -\frac{\sqrt{-6~A_6+6^{3/4}~\sqrt{-A_0~\sqrt{A_8}}}~\left( 1+e^{(-C~t+x)~D_1}~D_2\right) }{\sqrt{10}~\sqrt{A_8}~\left( -1+e^{(-C~t+x) D_1}~D_2\right) }\right) ~\rho ~e^{i(t ~\omega +\Theta -\kappa ~x)},\\&~D_1<0,~D_2<0,~A_0<0,~A_6<0,~A_8>0. \end{aligned} \end{aligned}$$

Thus, the corresponding Kink-type soliton solutions are obtained in the following form:

27 $$\begin{aligned} \begin{aligned}&u(x,t)=\left( \frac{\sqrt{-6 A_6+6^{3/4} \sqrt{-A_0 \sqrt{A_8}}} \left( 1-e^{(-C t+x) D_1} D_2\right) }{\sqrt{10} \sqrt{A_8} \left( 1+e^{(-C t+x) D_1} D_2\right) }\right) ~e^{i(t ~\omega +\Theta -\kappa ~x)},\\&\upsilon (x,t)=\left( \frac{\sqrt{-6 A_6+6^{3/4} \sqrt{-A_0 \sqrt{A_8}}} \left( 1-e^{(-C t+x) D_1} D_2\right) }{\sqrt{10} \sqrt{A_8} \left( 1+e^{(-C t+x) D_1} D_2\right) }\right) ~\rho ~e^{i(t ~\omega +\Theta -\kappa ~x)},\\&~~D_1<0,~D_2<0,~A_0<0,~A_6<0,~A_8>0. \end{aligned} \end{aligned}$$

## Graphical representation of the soliton solutions

Here, the characteristics of several obtained soliton solutions are illustrated through 2D and 3D graphical representations.

**Fig. 2:**
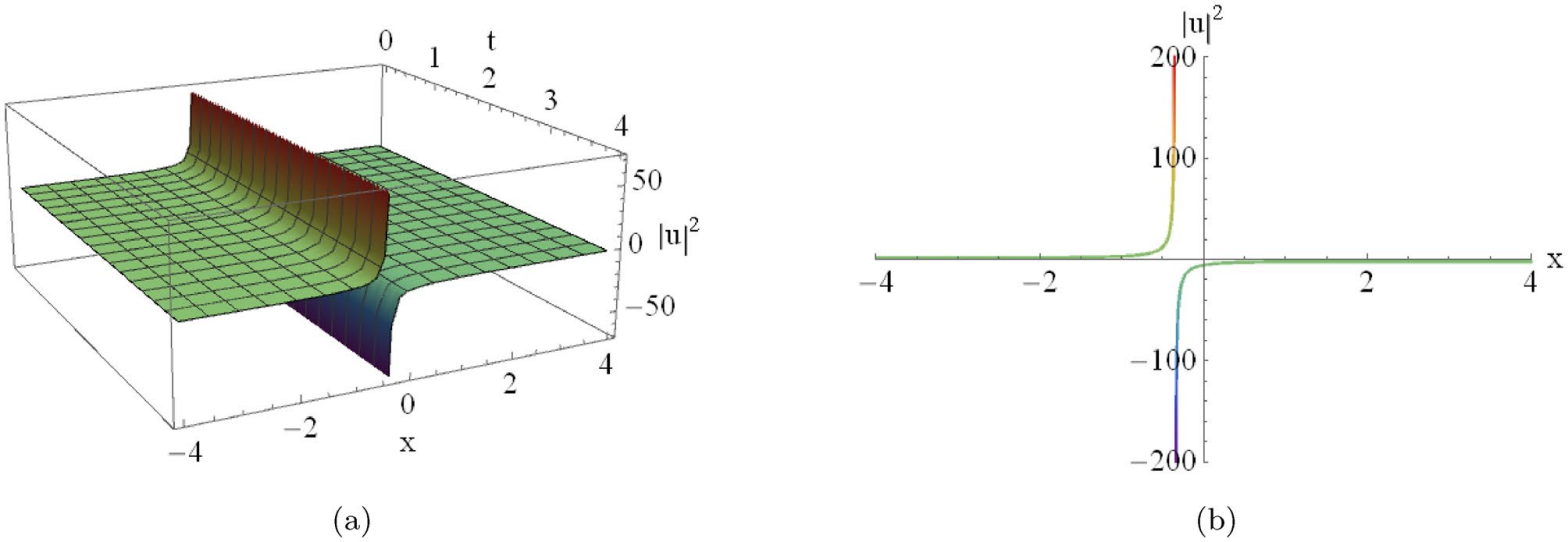
An illustrative depiction of of a singular soliton of Eqn. (18) by $D_0=2,~~D_1=-2, C=0,~~A_7=-2,~~A_8=-2.$ By changing the parameters $A_7$ and $A_8$, the graph will change as the following figures.

**Fig. 3:**
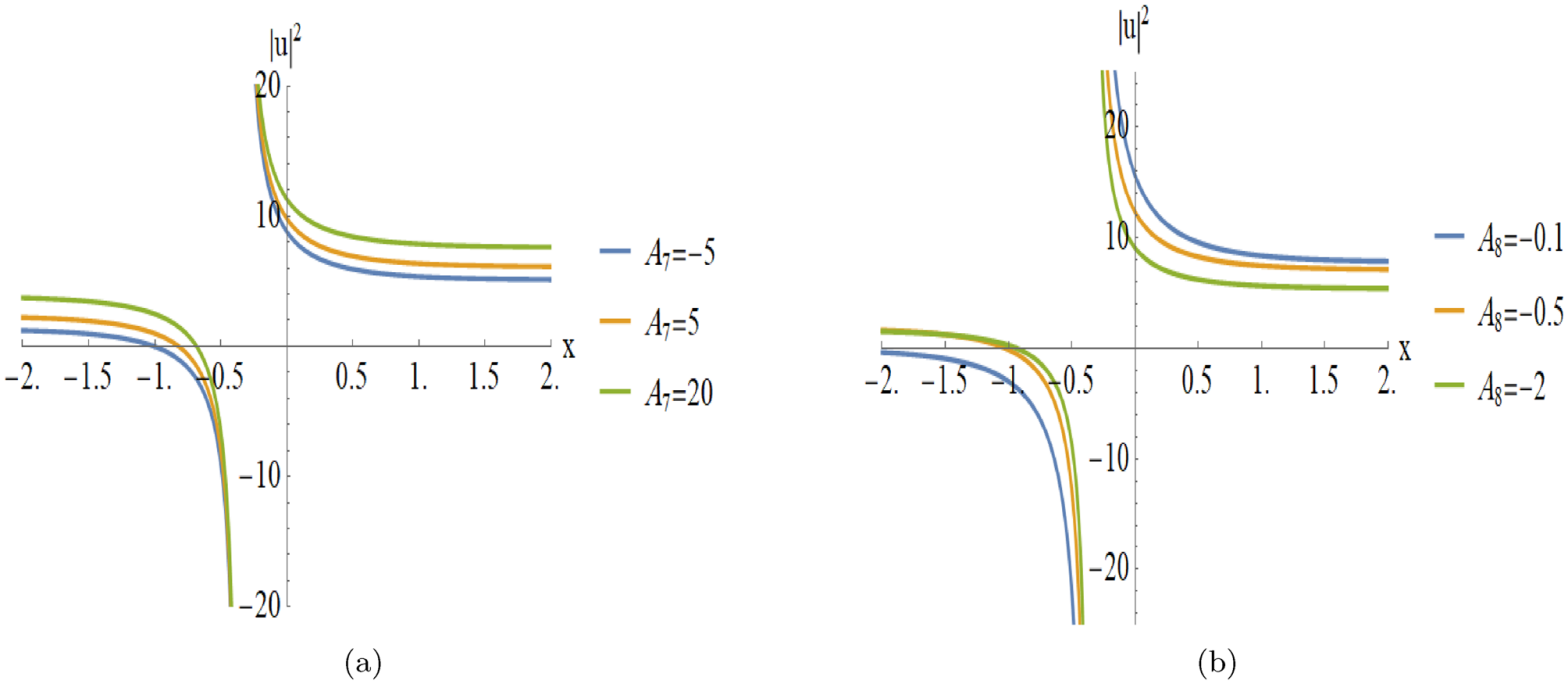
Parametric evolution of singular soliton solutions Eqn. (18) showing the impact of varying $A_7$ and $A_8$ on wave localization and background levels.

Figure 2(a) shows the three-dimensional profile of the singular soliton, while Fig. 3(b) depicts the corresponding two-dimensional surface representation obtained from Eqn. (18) for $D_0=2,\, D_1=-2,\, C=0,\, A_7=-2,$ and $A_8=-2$.

Figure 3 provides a detailed parametric analysis of the singular solutions. Specifically, Fig. 3(a) displays the structural changes under different values of $A_7$, while Fig. 3(b) demonstrates how the variation of $A_8$ modulates the soliton’s sharpness and localization.

**Fig. 4:**
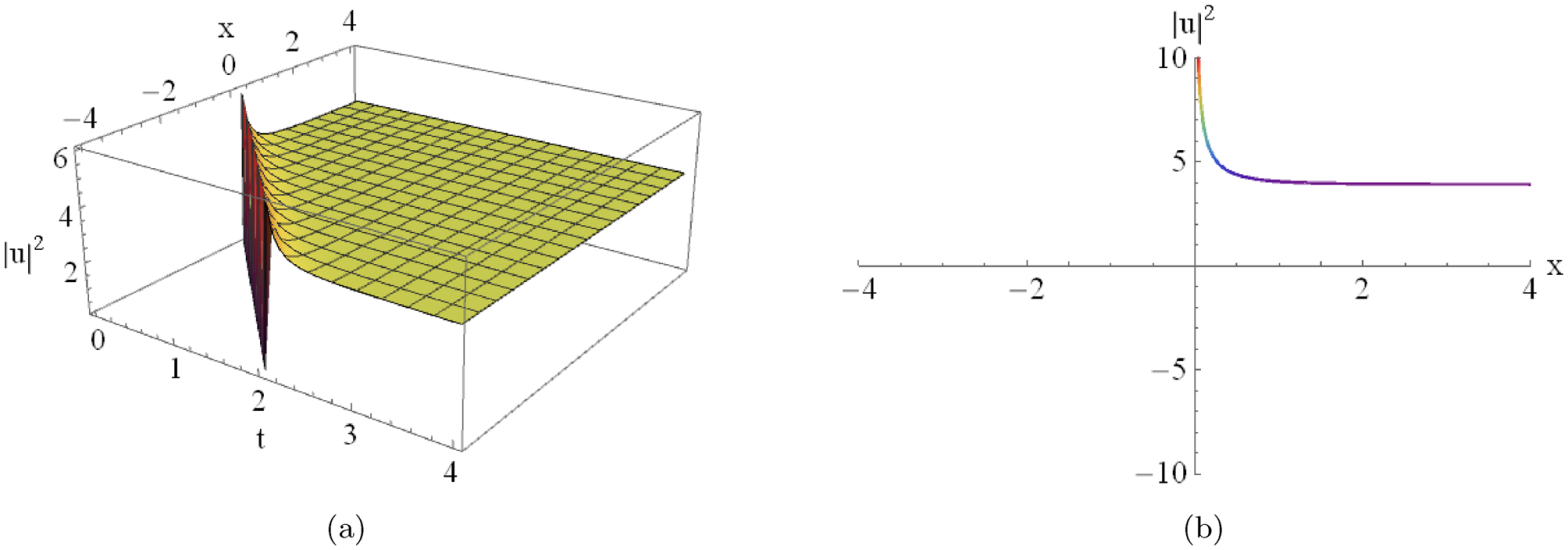
An illustrative depiction of of a singular soliton of Eqn. (20) by $D_0=-2,~~D_2=2,~~C=-2,~~A_7=2,~~A_8=-2.$ By changing the parameters $A_7$ and $A_8$, the graph will change as the following figures.

Figure 4(a) shows the three-dimensional profile of the singular soliton, while Fig. 4(b) depicts the corresponding two-dimensional surface representation obtained from Eqn. (20) for $D_0=-2,\, D_2=2,\, C=-2,\, A_7=2,$ and $A_8=-2$.

**Fig. 5:**
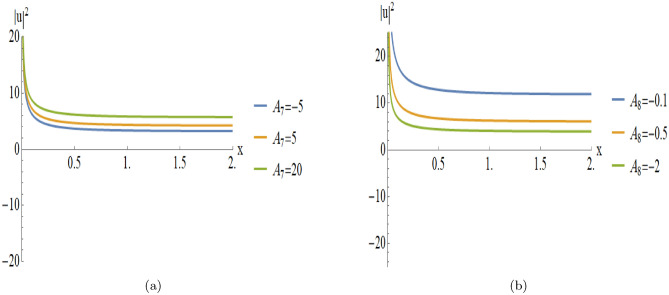
Parametric evolution of singular soliton solutions Eqn. (20) showing the impact of varying $A_7$ and $A_8$ on wave localization and background levels.

Figure 5 provides a detailed parametric analysis of the singular solutions. Specifically, Fig. 5(a) displays the structural changes under different values of $A_7$, while Fig. 5(b) demonstrates how the variation of $A_8$ modulates the soliton’s sharpness and localization.

**Fig. 6:**
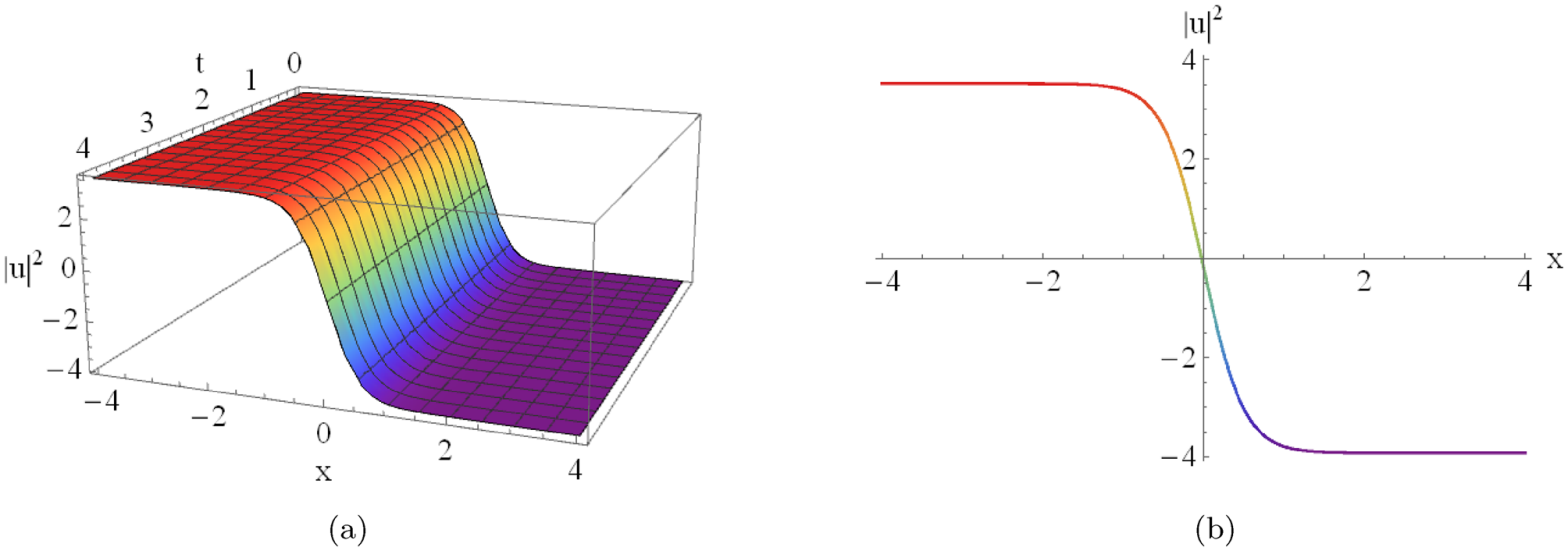
Graphical representation of an antikink-type soliton of Eqn. (23) by $D_0=-2,~~D_2=2,~~C=0,~~A_7=-2,~~A_8=-2.$ By changing the parameters $A_7$ and $A_8$, the graph will change as the following figures.

Figure 6(a) shows the three-dimensional profile of the antikink-type soliton, while Fig. 6(b) depicts the corresponding two-dimensional surface representation obtained from Eqn. (23) for $D_0=-2,\, D_2=2,\, C=0,\, A_7=-2,$ and $A_8=-2$.

**Fig. 7:**
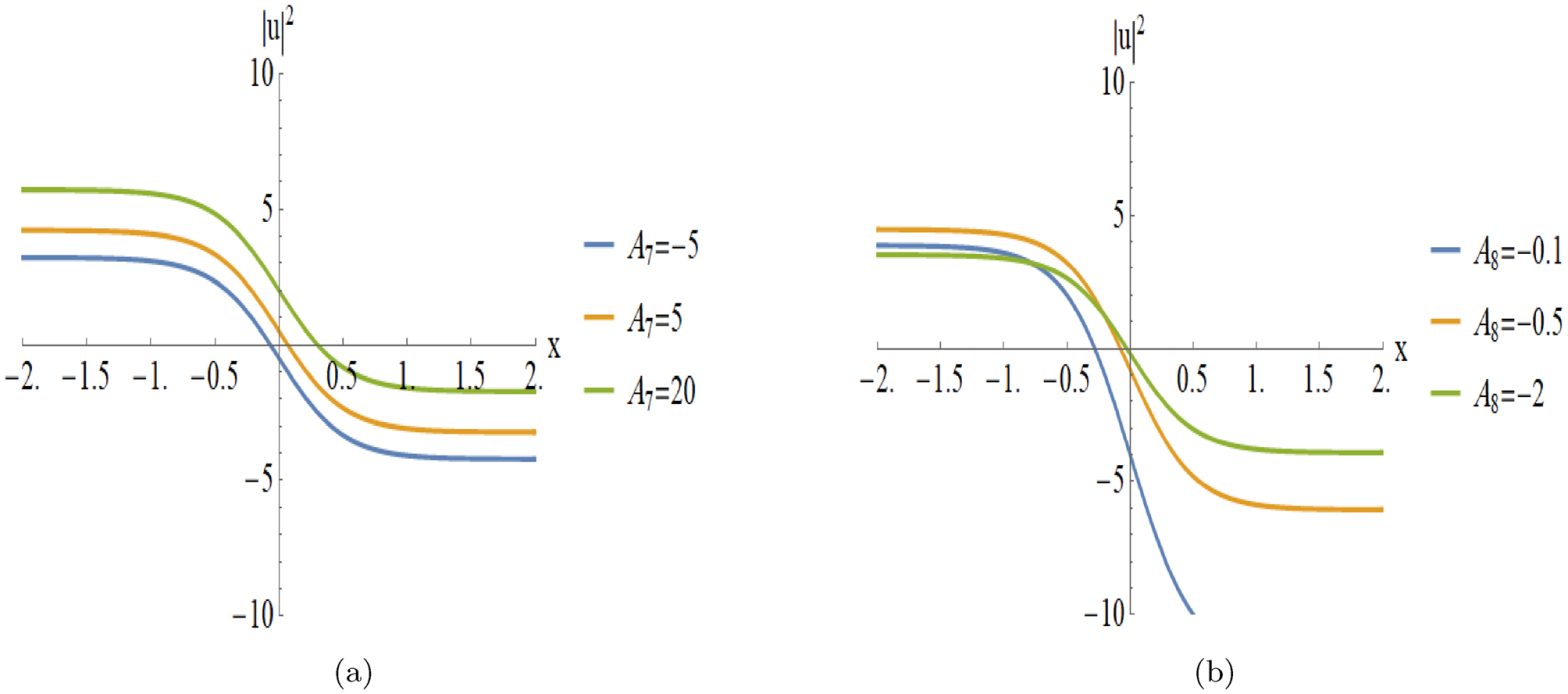
Parametric evolution of antikink-type soliton Eqn. (23) showing the impact of varying $A_7$ and $A_8$ on wave localization and background levels.

Figure 7 provides a detailed parametric analysis of the singular solutions. Specifically, Fig. 7(a) displays the structural changes under different values of $A_7$, while Fig. 7(b) demonstrates how the variation of $A_8$ modulates the soliton’s sharpness and localization.

**Fig. 8:**
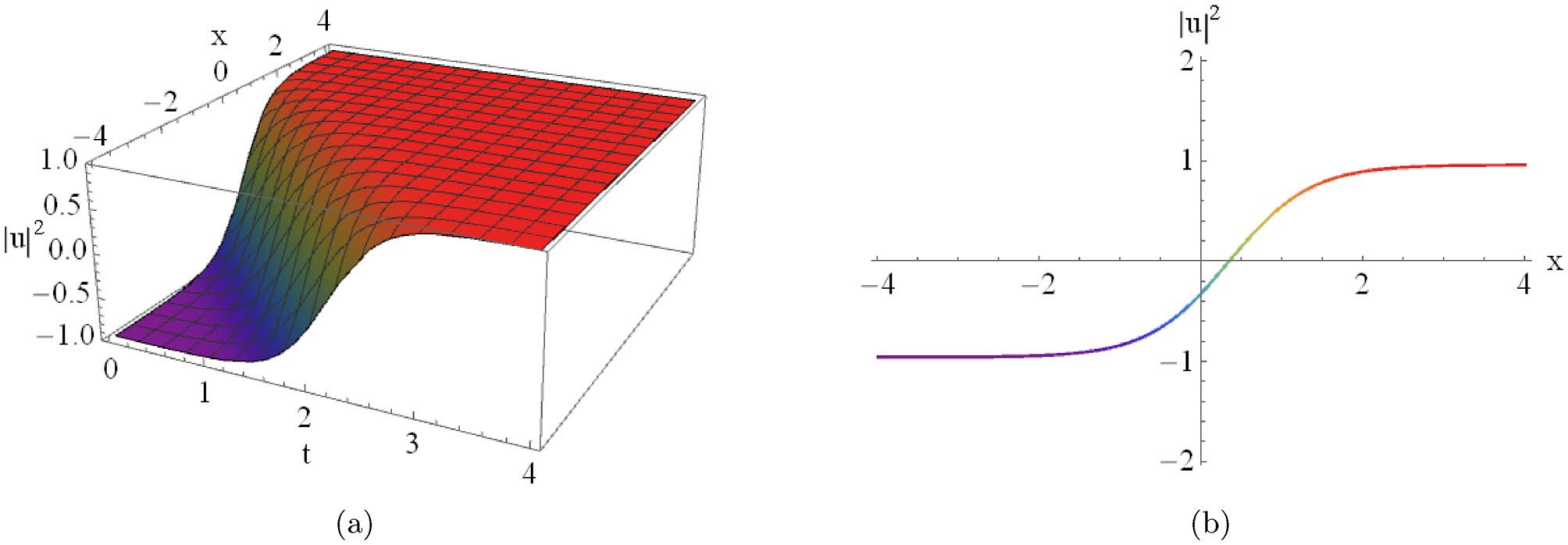
An illustrative depiction of a kink-type soliton solution of Eqn. (26) by $D_1=-2,~~D_2=-2,~~C=-2,~~A_0=-2,~~A_6=-2,~~A_8=2.$ By changing the parameters $A_0$, $A_6$ and $A_8$, the graph will change as the following figures.

Figure 8(a) shows the three-dimensional profile of the kink-type soliton solution, while Fig. 8(b) depicts the corresponding two-dimensional surface representation obtained from Eqn. (26) for $D_1=-2,\, D_2=-2,\, C=-2,\, A_0=-2,\, A_6=-2,$ and $A_8=2$.

**Fig. 9:**
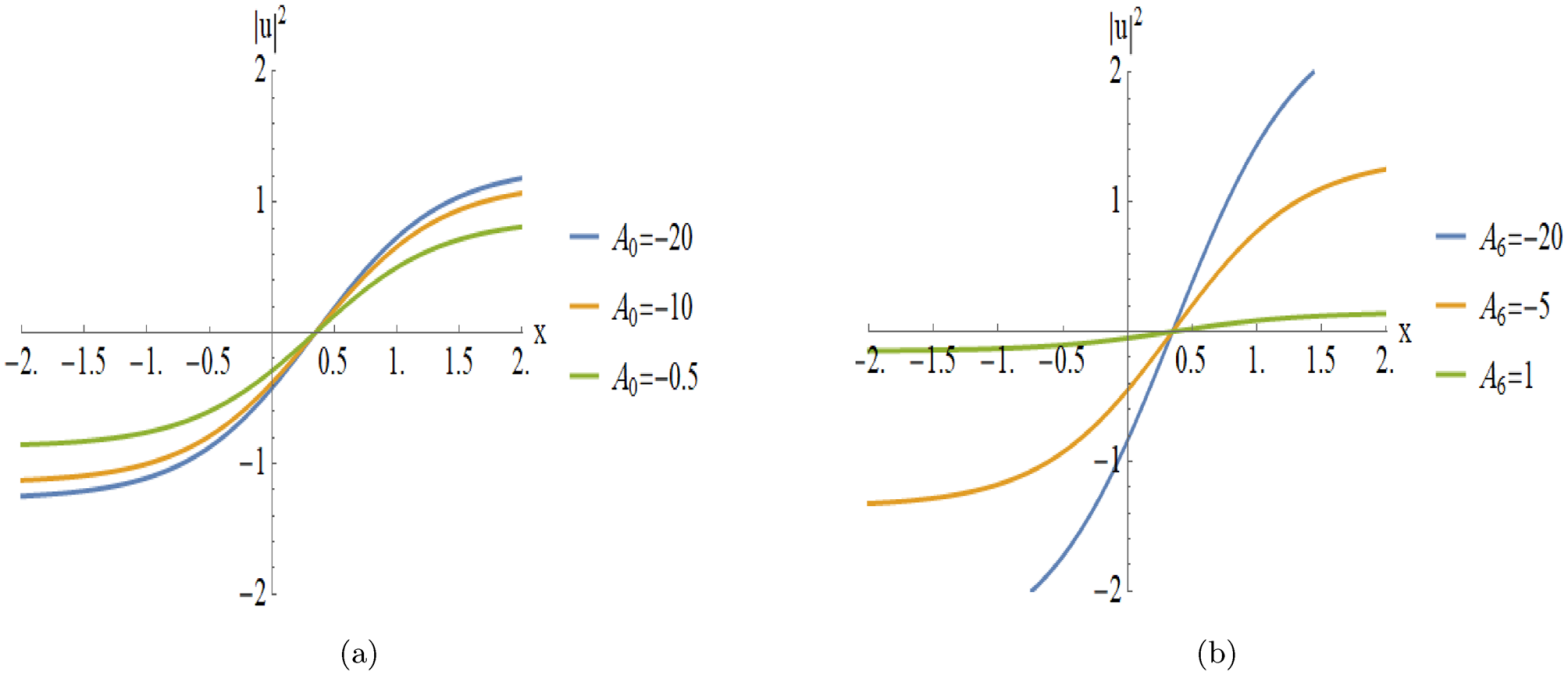
Parametric evolution of kink-type solutions Eqn. (26) showing the impact of varying $A_0$ and $A_6$ on wave localization and background levels.

**Fig. 10:**
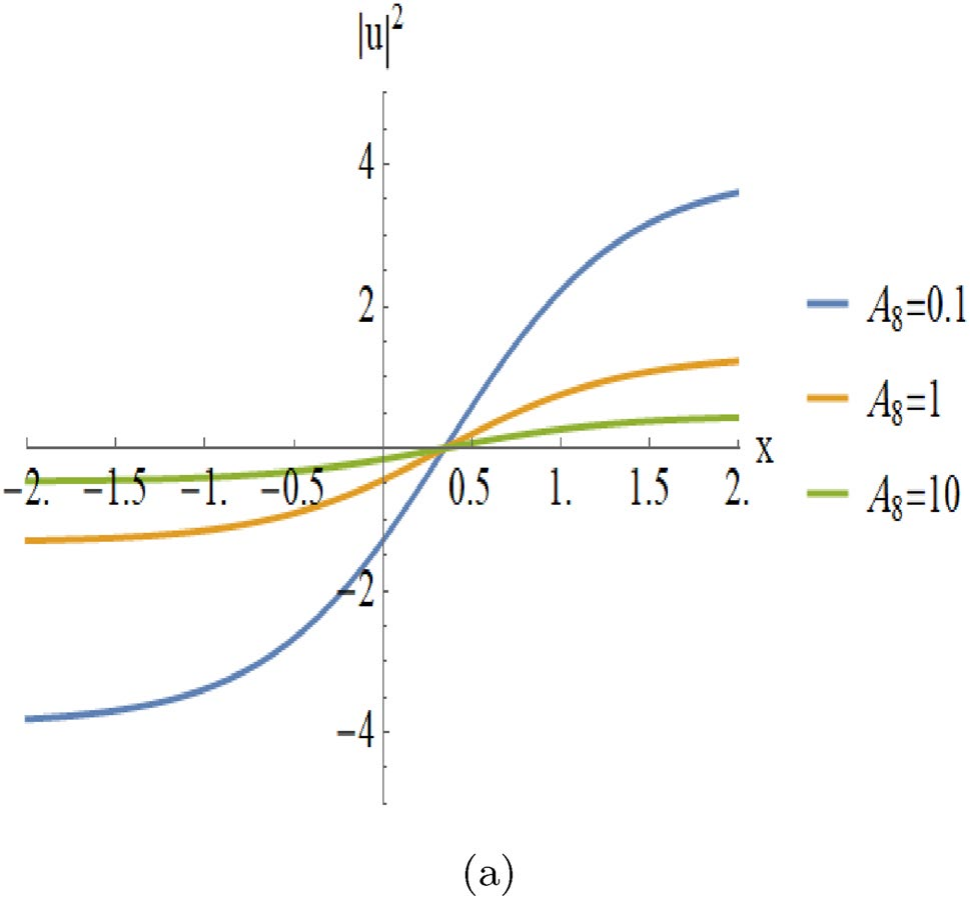
Parametric evolution of kink-type solutions Eqn. (26) showing the impact of varying $A_8$ on wave localization and background levels.

Figures 9, 10 provides a detailed parametric analysis of the singular solutions. Specifically, Fig. 9(a) displays the structural changes under different values of $A_0$, while Fig. 9(b) demonstrates how the variation of $A_6$ and Fig. 10(a) demonstrates how the variation of $A_8$ modulates the soliton’s sharpness and localization.

## Results and discussion

The derived soliton solutions demonstrate a fundamental dynamic equilibrium between higher-order nonlinear effects—specifically the Kudryashov-type refractive index—and the dispersive properties of the magneto-optic medium. This balance is the core mechanism that prevents dispersive broadening, allowing the pulses to maintain their localized profile during long-distance propagation.

This equilibrium manifests in diverse wave structures, as illustrated in our graphical analysis:

- **Singular Solitons (Figures** 2 & 5): These exhibit amplitude divergence at specific points, signaling the operational limits of the medium where nonlinear effects dominate. **First Case (Analysis of Fig.** 2): Fig. 3 illustrates the parametric variations for the solutions of Fig. 2:
  - **Varying** $$A_7$$ **(with** $$A_8 = -2$$**):**
    - **Asymptotic Level:** The profile shifts vertically as $A_7$ varies, indicating that $A_7$ modulates the background energy of the wave.
    - **Wave Structure:** The solution maintains its singular nature while the entire profile scales along the amplitude axis.
  - **Varying**$$A_8$$ **(with** $$A_7 = -2$$**):**
    - **Localization:** The soliton becomes sharper and more localized as $|A_8|$ increases, showing a higher concentration of energy near the singularity.
    - **Amplitude:** The peak intensity near the singular point increases significantly with larger absolute values of $A_8$.
  **Second Case (Analysis of Fig.** 4): Similarly, Fig. 5 demonstrates the influence of changing physical parameters on the base singular solution presented in Fig. 4:
  - **Varying** $$A_7$$ **(with** $$A_8 = -2$$**):**
    - **Asymptotic Level:** The profile shifts vertically as $A_7$ varies, which demonstrates that $A_7$ modulates the background energy level of the wave.
    - **Wave Structure:** The solution maintains its singular nature while the entire profile scales significantly along the amplitude axis.
  - **Varying** $$A_8$$ **(with** $$A_7 = 2$$**):**
    - **Localization:** The soliton becomes sharper and more localized as $|A_8|$ increases, indicating a higher concentration of energy near the singularity.
    - **Amplitude:** The peak intensity near the singular point increases substantially with larger absolute values of $A_8$.
- **Antikink-type Solitons (Fig.** 6): These exhibit a complementary topological structure, representing a monotonic transition from a higher to a lower asymptotic state. Figure 7 illustrates the parametric variations for the antikink profiles shown in Fig. 6:
  - **Varying** $$A_7$$ **(with** $$A_8 = -2$$**):**
    - **Asymptotic Level:** The wave profile undergoes a vertical displacement as $A_7$ varies, modulating the background intensity.
    - **Phase Transition:** The smooth monotonic transition remains stable, confirming robustness.
  - **Varying** $$A_8$$ **(with** $$A_7 = -2$$**):**
    - **Amplitude and Steepness:** Increasing $|A_8|$ leads to a sharper transition gradient.
    - **Localization:** Larger values of $|A_8|$ enhance the localization of the phase transition.
- **Kink-type Solitons (Fig.** 8): These illustrate a monotonic transition between two distinct asymptotic states. Figures (9– 10) illustrate the parametric variations:
  - **Varying** $$A_0$$ **(with** $$A_6 = -2, A_8 = 2$$**):**
    - **Asymptotic Shifting:** Variation in $A_0$ leads to a vertical displacement of the entire structure.
    - **Stability:** The wave maintains its topological form, proving stability.
  - **Varying** $$A_6$$ **(with** $$A_0 = -2, A_8 = 2$$**):**
    - **Structural Scaling:** $A_6$ significantly influences the vertical stretch of the kink.
    - **Gradient modulation:** $A_6$ acts as a scaling factor for the wave intensity.
  - **Varying** $$A_8$$ **(with** $$A_0 = -2, A_6 = -2$$**):**
    - **Steepness and Sharpness:** Small values of $A_8$ produce a steeper kink, whereas larger values flatten it.
    - **Switching Control:** $A_8$ is the primary parameter for controlling switching speed.

The stable propagation of these solitons is essential for the design of high-performance optical devices. By tuning parameters like nonlinear coefficients and dispersion, one can effectively control pulse shape and velocity. Beyond optical applications, these results offer insights into plasma waves and Bose–Einstein condensates.

The obtained soliton solutions have substantial implications for practical engineering in nonlinear photonics. Specifically, the stability of these pulse profiles is critical for high-speed optical communications, where they facilitate increased bandwidth and reduced signal loss. Furthermore, the ability to manipulate these solitons suggests a pathway for designing all-optical switches, potentially replacing electronic-to-optical conversion modules. Finally, given the focus on magneto-optic waveguides, these results offer a theoretical foundation for developing advanced photonic devices, such as optical isolators and sensors operating in magnetized environments.

## Conclusion

This study presented a detailed analytical investigation of soliton propagation within magneto-optic waveguides, governed by a coupled nonlinear Schrödinger system featuring Kudryashov-type nonlinearities. By overcoming the limitations of standard modeling techniques through the systematic application of the ISEM, this work successfully uncovers a rich family of exact analytical solutions—including antikink-type, kink-type, singular, rational, exponential, and singular periodic waves—which were previously unexplored in this complex configuration. These results demonstrate how higher-order effects, such as self-steepening and dispersion, interact with magneto-optic parameters to fundamentally govern the soliton’s amplitude, phase modulation, and stability.

The findings provide a theoretical framework for future studies, including extensions to fractional-order derivatives, parity–time symmetric media, and systems involving stochastic perturbations. Furthermore, validating these analytical results via numerical techniques—such as split-step Fourier methods—and exploring their experimental realization in magneto-optic fibers and metamaterials are key avenues for bridging the gap between theory and practical engineering.

Ultimately, these soliton solutions offer significant utility in modern nonlinear photonics and telecommunications, particularly in:

- **High-Speed Optical Communications:** By ensuring pulse stability during long-distance transmission, these solutions contribute to increased bandwidth and reduced signal loss in fiber optic networks.
- **All-Optical Switching:** The ability to manipulate soliton profiles, as demonstrated by our analytical results, establishes a theoretical basis for designing all-optical switches that eliminate the need for electronic-to-optical conversion.
- **Magneto-Optic Device Development:** The model’s focus on magneto-optic waveguides provides critical insights for designing photonic devices that operate in magnetized environments, including optical isolators and sensors.

## Data Availability

The datasets used and/or analyzed during the current study are available from the corresponding author upon reasonable request
